# Deciphering Rind Color Heterogeneity of Smear-Ripened Munster Cheese and Its Association with Microbiota

**DOI:** 10.3390/foods13142233

**Published:** 2024-07-16

**Authors:** Amandine J. Martin, Anne-Marie Revol-Junelles, Jérémy Petit, Claire Gaiani, Marcia Leyva Salas, Nathan Nourdin, Mohammed Khatbane, Paulo Mafra de Almeida Costa, Sandie Ferrigno, Bruno Ebel, Myriam Schivi, Annelore Elfassy, Cécile Mangavel, Frédéric Borges

**Affiliations:** 1 Laboratoire d’Ingénierie des Biomolécules (LIBio), Université de Lorraine, F-54000 Nancy, France; amandine.martin@univ-lorraine.fr (A.J.M.); anne-marie.revol@univ-lorraine.fr (A.-M.R.-J.); jeremy.petit@univ-lorraine.fr (J.P.); claire.gaiani@univ-lorraine.fr (C.G.); marcia.leyva-salas@univ-lorraine.fr (M.L.S.); nathan.nourdin1@etu.univ-lorraine.fr (N.N.); mohammed.khatbane@univ-lorraine.fr (M.K.); myriam.schivi@univ-lorraine.fr (M.S.); annelore.elfassy@univ-lorraine.fr (A.E.); cecile.mangavel@univ-lorraine.fr (C.M.); 2 Instituto Federal Catarinense, Campus Concórdia, Rodovia SC 283, Km 17, Concórdia 89703-720, SC, Brazil; paulo.almeida@ifc.edu.br; 3 INRIA Nancy—Grand Est, Institut Elie Cartan de Lorraine (IECL), Equipe BIology, Genetics and Statistics (BIGS), Université de Lorraine, F-54000 Nancy, France; sandie.ferrigno@univ-lorraine.fr; 4 Laboratoire Réactions et Génie des Procédés, Université de Lorraine, CNRS UMR 7274, F-54518 Vandoeuvre les Nancy, France; bruno.ebel@univ-lorraine.fr

**Keywords:** cheese, color, microbiota, heterogeneity, image analysis

## Abstract

Color is one of the first criteria to assess the quality of cheese. However, very limited data are available on the color heterogeneity of the rind and its relationship with microbial community structure. In this study, the color of a wide range of smear-ripened Munster cheeses from various origins was monitored during storage by photographic imaging and data analysis in the CIELAB color space using luminance, chroma, and hue angle as descriptors. Different levels of inter- and intra-cheese heterogeneity were observed. The most heterogeneous Munster cheeses were the darkest with orange-red colors. The most homogeneous were the brightest with yellow-orange. K-means clustering revealed three clusters distinguished by their color heterogeneity. Color analysis coupled with metabarcoding showed that rinds with heterogeneous color exhibited higher microbial diversity associated with important changes in their microbial community structure during storage. In addition, intra-cheese community structure fluctuations were associated with heterogeneity in rind color. The species *Glutamicibacter arilaitensis* and *Psychrobacter nivimaris/piscatorii* were found to be positively associated with the presence of undesirable brown patches. This study highlights the close relationship between the heterogeneity of the cheese rind and its microbiota.

## 1. Introduction

Color is one of the most important attributes influencing consumer perceptions of quality, as it provides a clue for the evaluation of cheese characteristics such as flavor or ripeness [1]. It is one of the most important selection criteria in a consumer’s purchasing decision. Color is also an essential marker of the identity of smear-ripened cheeses. For instance, in the case of Munster, a smear-ripened cheese of the red-smear sub-family, according to the Protected Designation of Origin (PDO), the rind should have a color ranging between ivory-beige and orange-red [2].

The color of cheeses can be evaluated within the L*a*b* colorimetric system [3]. This system has been defined by the “Commission internationale de l’éclairage” in three cartesian dimensions and integrates the light source and object color spectrum into the reflectance spectrum [4]. L* axis corresponds to luminance, whereas a* and b* correspond to chromatic coordinates on the green–red and blue–yellow axes, respectively. Other color metrics, such as the hue and chroma, can be extracted from L*a*b*. The coordinates on the a*b* plane determines the hue angle h that gives the predominant wavelength of the color. For instance, an angle of 0° corresponds to red, an angle of 90° corresponds to yellow, an angle of 180° corresponds to green, and an angle of 270° corresponds to blue. The chroma, C*, accounts for the saturation or the color purity. Spectrocolorimetry analyses of five different PDO red-smear soft cheeses have shown that there is an inter-cheese color heterogeneity between cheeses from different PDO and among cheeses processed within the same PDO area [3]. The color of the rind of the Munster cheeses from six French producers showed the highest inter-cheese heterogeneity, with some cheeses being more yellow and others more reddish [3].

The yellow-orange pigments responsible for smear-ripened cheese color can be produced by a wide range of microorganisms belonging to the phylum *Actinomycetota*, and more specifically to the genera *Brevibacterium* and *Glutamicibacter* [5,6,7]. The orange color of red-smear cheeses is due to the synthesis of at least two families of pigments, carotenoids, and porphyrins, produced by the microbial flora during ripening [8,9]. *Brevibacterium linens* and *Glutamicibacter arilaitensis* are able to produce a large panel of carotenoid pigments responsible for color development at the cheese surface [6,10]. Carotenoids are secondary metabolites belonging to the terpenoid metabolism that play a role in oxidative stress resistance [11]. Depending on their structure, carotenoids can be yellow, orange, red, or purple [12]. A wide range of pigments are produced by *G. arilaitensis* [13], including reddish-brown [9] or pink porphyrins [14]. The coloring capacity of pigment producers can be influenced by biotic interactions. Yeasts can indeed have an impact on cheese color thanks to their deacidification activity, which creates favorable growth conditions for pigment-producing bacteria. Additionally, the yeast species involved in the deacidification process have a notable qualitative impact on color [14,15,16,17]. Moreover, abiotic factors such as temperature, light, dissolved oxygen, salt, curd type, and growth substrates are important for bacterial development and act on pigment production, thereby influencing the color of the rind [3,16,18,19,20,21]. Although variations in the microbial communities colonizing cheese are described to influence important aspects of cheese ripening [22], limited data are available about the role of communities and their structure on cheese color.

Structured environments are subject to heterogeneity, which is defined as the variation in biotic and abiotic conditions across space and time [23]. In structured environments, the assembly of community gives rise to biodiversity patterns consisting in a heterogeneous distribution of microorganisms at the temporal and spatial scales [24]. At the temporal scale, patterns of microbial successions can be observed in smear-ripened cheeses: during the beginning of ripening, yeasts colonize the cheese surface thanks to lactate utilization, resulting in an increase in pH, thus creating favorable conditions for the development of a complex bacterial community [25]. At the spatial scale, the cheese microbiota of washed–ripened cheeses can vary largely between processing facilities [26] but also between cheeses produced in the same dairy plant [27]. Even at the scale of a single cheese, the distribution of microorganisms is highly heterogeneous between the core and the rind: the core is dominated by lactic acid bacteria, while the rind can be colonized by a wide range of yeasts, molds, and aerobic bacteria [28]. Despite all the studies carried out to understand the spatiotemporal variability of the microbiota, there are very limited data linking this variability to the color heterogeneity of the cheese surface at different scales, including the intra-cheese scale.

The aim of this study was to investigate the color heterogeneity of the rind of the PDO Munster cheese and its relationship with microbial community structure, as well as with the pH and salt concentration. To do so, a large set of commercial Munster cheese samples produced by diverse manufacturing plants was used to investigate the evolution of the color within the same PDO area. Two other sets of samples were obtained from a manufacturing plant that regularly produces cheese with color defects during cheese storage (from packaging to the best-before date). These color defects can be characterized by the appearance of patches with different colors perceptible to the human eye. The two sets of samples obtained from this plant were used to investigate the relationship with biotic and abiotic parameters at different spatial and temporal scales. These data could be used by cheesemakers to tune the cheese manufacturing process in order to achieve a desired heterogeneity level in the produced cheeses.

## 2. Materials and Methods

### 2.1. Sampling and Sample Preparation

A sample set of 33 PDO Munster cheeses, labeled from M01 to M33, originating from 13 different production units, was used to investigate the spatiotemporal evolution of rind color over time. The cheeses were bought on the market in order to cover a wide range of producers and sizes. The cheeses were monitored for 40 days (18 days before and 22 days after the best-before date) from April to June 2022. Only the side visible when the cheeses were unpacked was photographed, as the other side was altered due to the direct contact with the sample pedestal in the lightbox. The cheeses were stored at 4 °C in the dark without humidity control between image captures and kept in the original packaging. For each cheese, from 7 to 11 photographs were taken.

Another sample set of 8 Munster cheeses from 2 randomly selected batches was obtained from factory 1. Cheeses from batches 1 and 2 were labeled M34 to M37, and M41 to M44, respectively. They were used to investigate the links between color heterogeneity, community structure, pH, and salt concentration. Cheeses from batches 1 and 2 were sampled from 2 different lots produced in the same factory on 21 February 2023 and 7 March 2023, respectively. The cheeses were analyzed immediately after packaging (D-45) and 45 days after the best-before date (D0). The cheeses were stored at 4 °C in the dark without humidity control between these 2 time points in their original packaging. Only 1 side of each cheese was monitored: 1 half of the monitored side was analyzed at D-45, the other half at D0. For each half of each monitored cheese side, a photograph was taken, and pH and salt were subsequently measured by using direct contact approaches, without taking samples. Then, each half of each monitored cheese side was scraped with a scalpel for enumeration, and DNA was extracted for metabarcoding analysis. To do so, the resulting sampled rinds were weighted and resuspended in 200 mL of citrate buffer (20 g·L^−1^ of trisodium citrate dihydrate, NORMAPUR^®^, Singapore), pre-heated at 50 °C in a stomacher bag and mixed in a homogenizer stomacher. The solubilized cheese samples were kept in an ice bath until enumeration and DNA extraction. 

A third set of samples consisting of 2 Munster cheeses (M48 and M49), selected for their particular intra-cheese spatial heterogeneity of color, was obtained from factory 1 on 5 May 2022. Cheeses M48 and M49 were stored at the laboratory in their original packaging at 4 °C in the dark without humidity control until analysis and were sampled 14 days before and 28 days after the best-before date, respectively. After image capture, from 3 to 6 samples were taken in each of the 2 types of color zones, which were defined by the cheesemaker as “acceptable” or “undesirable”. Approximately 10 mg rind (corresponding to approximately 0.2 cm^2^) was sampled with a sterile scalpel and used for DNA extraction.

### 2.2. Imaging Setup

Cheeses were photographed in a standardized lightbox (STARBLITZ, Montreuil, France, cube of 40 cm side), equipped with 120 LEDs for backlighting, where light was set at the maximum intensity. This equates to 8.35 ± 0.12 lux in the photographed area. The images were taken with a Nikon D5600 digital camera (Nikon Corp, Tokyo, Japan) equipped with an AF-S DX NIKKOR 18–140 mm f/3.5–5.6 G ED VR (Nikon Corp, Tokyo, Japan) set to 30 mm focal length. The digital camera was set to a high depth of field (F/11) to obtain sharp images regardless of the height of the cheese sample, a low sensor value (ISO 100) to limit numerical noise, and 1/30 s exposure. The camera was positioned vertically above the samples, and the pictures were taken at a resolution of 4496 × 3000 pixels and saved in JPG format. The white balance was set once by using a Novoflex ZEBRA XL gray card (Westerkappeln, Germany, 21 cm × 30 cm) positioned in the camera field of the lightbox and recorded in the digital camera to be used throughout the experiment. The images were imported and processed into R v 4.3.1 [29]. More precisely, the images in JPG format were imported and compressed 3 times with the package imager [30]. Each image was imported in the red, green, blue (RGB) color space, where each pixel was associated with particular RGB values in a digital image [31]. The original image pixels were transformed from RGB to the CIE L*a*b* color space using the R package schemr v0.2.0 [32]. In this uniform system, L* is the luminance coefficient (ranging from black = 0 to white = 100), a* is the green-magenta balance (green for negative values ranging from −100 to 0, and magenta for positive values ranging from 0 to +100), and b* is the blue-yellow balance (blue for negative values ranging from −100 to 0, and yellow for positive values ranging from 0 to +100). The a* and b* values were used to calculate the chroma (C*) following the formula C* = $\sqrt{a{*}^{2}+{b*}^{2}}$, and the hue angle (h) with the formula h = tan^−1^ ($\frac{b*}{a*}$), thanks to a custom R script. C* corresponds to the intensity of a color (ranging from 0 to 100), while the h corresponds to the hue angle in the color wheel (0° to 360°).

### 2.3. Rind pH and Salt Concentration Measurements

The 8 Munster cheeses from batches 1 and 2 (from M34 to M37, and from M41 to M44, respectively) were subjected to pH and salt measurements. For each half of the investigated cheese side, 10 pH measurements at different locations of the rind were performed by using a pH meter METTLER TOLEDO FiveEasy (Greifensee, Switzerland) coupled to a surface sensor (VWR 662-1763, Leuven, Belgium). Similarly, 10 measurements of salt concentration (in arbitrary units) were performed for each half of the cheese side by using a portable salt meter (PAL-SALT PROBE, ATAGO, Tokyo, Japan). Before measurements, the salt meter was calibrated with deionized water and 2.5% NaCl water solution. 

### 2.4. Bacterial and Yeast Mold Enumeration

Each rind suspension of batches 1 and 2 (from M34 to M37, and from M41 to M44, respectively) was serially diluted tenfold in a tryptone-salt solution (9.5 g·L^−1^, Biokar Diagnostics, Beauvais, France) and homogenized by vortexing. One hundred microliters of diluted suspensions was used to inoculate agar culture medium dispensed in Petri dishes in order to manually count colonies for enumeration after cultivation. The medium used was the Plate Count Agar Milk Salt medium (PCAMS), which consisted of plate count agar (PCA, BIOKAR diagnostics, Beauvais, France) supplemented with 1 g·L^−1^ skim milk powder (Millipore, Merck, Darmstadt, Germany) and 10 g·L^−1^ NaCl (VWR, Leuven, Belgium) [33]. For bacterial enumeration, PCAMS medium was supplemented with 2.28 mg.L^−1^ natamycine. For yeast and mold enumeration, PCAMS was supplemented with 50 mg.L^−1^ chloramphenicol. Plates were incubated in triplicate at 20 °C for 72 h.

### 2.5. DNA Extraction

For samples of batches 1 and 2 (from M34 to M37, and from M41 to M44, respectively), a volume of solubilized rind (in citrate buffer) corresponding to 10 mg of rind was diluted in 10 mL phosphate-buffered saline (PBS, 0.01 M phosphate buffer, 0.0027 M potassium chloride, 0.0137 M sodium chloride, pH 7.4 at 25 °C, Sigma, St. Louis, MO, USA). For samples M48 and M49, approximately 10 mg rind was diluted in 10 mL PBS. After the diluted samples were centrifugated at 6400× *g* for 10 min at 4 °C, the supernatants were discarded, and the pellets were washed twice with 10 mL PBS. Each pellet was subsequently resuspended in 1 mL of PBS and stored at −20 °C until use for DNA extraction. 

The DNA extraction was performed using the NucleoSpin Food^®^ kit (Macherey-Nagel, Düren, Germany) following the method previously described [34]. Each sample was centrifuged at 10,000× *g* for 10 min, and the supernatant was discarded. The pellet was then suspended in 550 µL of lysis buffer preheated at 65 °C, and 10 µL of 10 mg·mL^−1^ proteinase K (Macherey-Nagel, Düren, Germany) was added. The homogenized mixture was then incubated at 65 °C for 3 h. Subsequently, 10 µL of 20 mg·mL^−1^ RNase (Sigma) and between 200 and 300 mg of sterile, UV-treated glass beads (150–212 µm diameter, Sigma) were added, and the sample was shaken at 3200 rpm on a Vortex Génie 2 horizontal agitator (Scientific Industries, New York, NY, USA) for 1 h at room temperature. After centrifugation at 10,000× *g* for 10 min, the supernatant was used for DNA purification according to the manufacturer’s instructions. Quality control of the extracted DNA was performed by spectrophotometry (Nanodrop 2000c, Thermo Fisher Scientific, Waltham, MA, USA) and agarose gel electrophoresis. 

### 2.6. Amplicon Sequencing

To analyze the bacterial community structure, polymerase chain reaction (PCR) amplification targeting the V3–V4 region of the 16S rRNA gene was performed. The primers used for PCR consisted of the universal targeting sequences TACGGRAGGCWGCAG (forward) and TACCAGGGTATCTAATCCT (reverse) [35], to which adapter sequences were added at the 5′ extremity (Table 1). To 37.3 µL DNA-grade water were added 5 µL AccuPrime^TM^ Buffer II (Invitrogen, Carlsbad, CA, USA), 0.42 µL of each forward primer at 10 µM, 1.25 µL reverse primer at 10 µM, 0.2 µL AccuPrime^TM^ Taq DNA polymerase High Fidelity (Invitrogen, Carlsbad, CA, USA), and 5 µL of 20 ng·µL^−1^ DNA. After an initial denaturation step at 94 °C for 3 min, 35 PCR cycles were performed as follows: 30 s denaturation at 94 °C, 1 min hybridization at 63.6 °C, and 1 min elongation at 68 °C. A final elongation step was performed at 68 °C for 10 min. Miseq V2 Illumina sequencing was conducted by ADNid (Monferriez-sur-Lez, France), providing 2 × 250 bp paired-end reads. 

To analyze the fungal community structure, a PCR amplification targeting the ITS region was performed. The primers used for PCR consisted of the universal targeting sequences CTTGGTCATTTAGAGGAAGTAA (forward) [36] and GCTGCGTTCTTCATCGATGC (reverse) [37], to which adapter sequences were added at the 5′ extremity (Table 1). To 37.8 µL DNA-grade water were added 5 µL AccuPrime^TM^ Buffer II (Invitrogen, Carlsbad, CA, USA), 0.33 µL of each forward primer at 10 µM, 1 µL of reverse primer at 10 µM, 0.2 µL AccuPrime^TM^ Taq DNA polymerase High Fidelity, and 5 µL of 20 ng·µL^−1^ DNA extract. After an initial denaturation step at 94 °C for 3 min, 30 PCR cycles were performed as follows: 30 s denaturation at 94 °C, 45 s hybridization at 50 °C, and 90 s elongation at 68 °C. A final elongation step was performed at 68 °C for 10 min. Miseq V2 Illumina sequencing was conducted by ADNid (Monferriez-sur-Lez, France), providing 2 × 250 bp paired-end reads. 

### 2.7. Sequence Analysis

The sequencing reads were analyzed using FROGS tools [38] on the Galaxy Migale platform. The 16S RNA gene and ITS paired-end reads were merged with a maximum mismatch rate of 10% in the overlap region. Merged reads that did not match the expected length (600 pb) or contained ambiguous bases (N) were discarded. After dereplication, sequences were clustered with the fastidious option in SWARM, a single-linkage clustering algorithm using a local clustering threshold (aggregation distance of 1 in the present work) [39]. After chimeras were removed, the reads representing less than 0.005% were discarded, and the filtered data were used for taxonomic affiliation by using BLAST and the 16S EZBioCloud 52018 database [40] for 16S rRNA sequences, and the ITS UNITE 7.1 [41] for ITS sequences. The read counts were filtered by conversion to 0 when the value was below 10. The abundance and multi-affiliation tables were then imported into R v 4.3.1 [29] for further analysis using the R package phyloseq v1.46.0 [42], ape v5.8 [43], microbiome v1.2.4.0 [44], vegan v2.6-6.1 [45], DECIFER v 2.30.0 [46], phangorn v 2.11.1 [47], and Biostrings v 2.70.3 [48]. 

### 2.8. K-means Clustering

K-means clustering was used to classify the 33 PDO Munster cheeses on the basis of the color of their rinds. To do so, for every investigated colorimetric parameter L*, C*, and h, the mean and the standard deviation were calculated. For each mean and standard deviation of the 3 L*, C*, and h variables, 2 linear models were built, 1 corresponding to the period before the best-before date, and the other to the period after the best-before date. Model parameters (slope and intercept) were extracted, and the resulting overall 24 terms (3 color variables × 2 statistical metrics × 2 time periods × 2 model parameters = 24) were used for clustering. K-means clustering was chosen because of the large number of quantitative variables. 

The optimal number of clusters was determined by the Silhouette method. A principal component analysis (PCA) was performed to visualize the groups, and the quality of partitioning was estimated thanks to the Silhouette width metrics by using the package factoextra v1.0.7 [49]. Data processing and statistical analyses were performed using R v 4.3.1 [29].

### 2.9. Statistical Analysis

Statistical analyses were conducted using R v 4.3.1 [29]. Pearson’s product-moment correlation tests were employed to assess correlations, given that the data were normally distributed. Simple linear regressions were utilized to further explore relationships between quantitative variables. When not otherwise specified, two-way repeated measures analysis of variance (ANOVA) was used. Post hoc multiple pairwise comparisons were performed with the adjusted Bonferroni multiple testing correction method. Differences were considered statistically significant if the *p* value was less than 0.05. Additionally, linear discriminant analysis effect size (LEfSe) was conducted on the microbial relative abundance data using the microbiomeMarker v1.8.0 package [50], with an LDA score cutoff of 4. 

## 3. Results

### 3.1. Color Palette of the PDO Munster Rind Cheese

**Figure 1:**
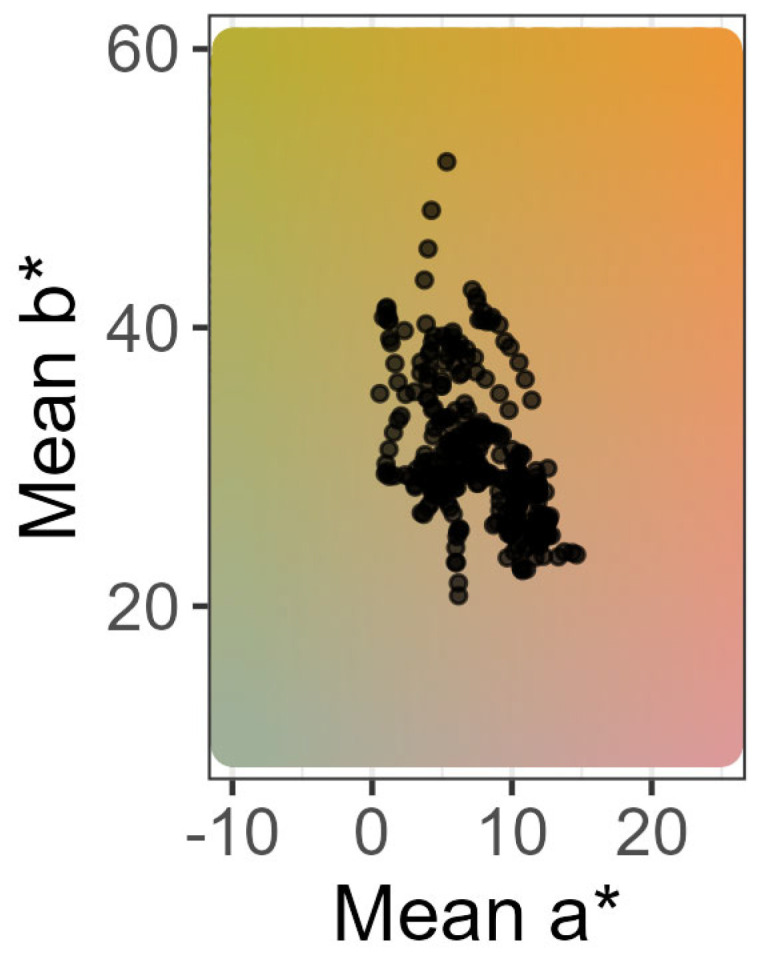
Color distribution of the rind of 33 PDO Munster cheeses (M1 to M33) in the a*b* plane. One point represents the mean calculated from the values for each pixel from one single image. All means calculated from the set of 316 images obtained from the monitoring of each cheese are represented. The values are shown at an L* value of 70.

To investigate the color of PDO Munster cheese, standardized images of 33 Munster cheeses, sampled from different factories and farms and processed from raw or pasteurized milk (Table S1), were taken during storage at 4 °C for 40 days, from 18 days before to 22 days after the best-before date, leading to a dataset comprising 316 photographs. Image analysis revealed that the mean a* values (green-magenta balance) of Munster cheeses (Figure 1) ranged from 0.6 to 14.9, and mean b* values (blue-yellow balance) were even more variable, with values ranging from 20.8 to 51.9, indicating that Munster cheeses tend to be more yellow than red. Mean color saturation C* ranged between 21.9 and 52.3 and mean hue angles between 58.4° and 88.8°, which corresponds to a range of colors from yellow-orange to orange-red. The luminance of Munster cheeses ranged from 57.3 to 75.2. In conclusion, a high color variability of Munster was observed, ranging from yellow-orange to orange-red.

### 3.2. Color Heterogeneity of the PDO Munster Cheese Rinds

**Figure 2:**
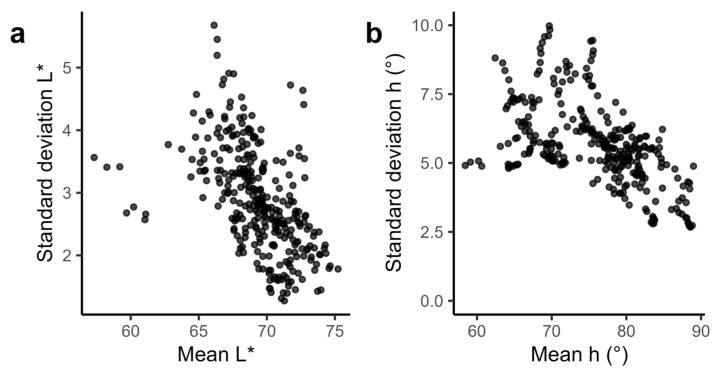
Relationship between the mean and the standard deviation of the luminance L* and the hue angle h. One single point represents the L* (**a**) and the h (**b**) mean and standard deviation values calculated from the overall pixels extracted from images taken over time. The data obtained for the 33 monitored PDO Munster cheeses (M1 to M33) are represented.

The standard deviations of L*, C*, and h colorimetric parameters were used as metrics of color heterogeneity, with a high standard deviation representing a high color heterogeneity. The images of the 33 monitored cheeses revealed a negative correlation between the mean luminance and the standard deviation of luminance (Figure 2a, r = −0.43, *p* = 0.015, Pearson’s product-moment correlation—PPMC calculated at the best-before date), indicating that the higher the luminance value, the lower the associated standard deviation. There was also a negative correlation between the mean hue angle and the corresponding standard deviation (Figure 2b, r = −0.56, *p* = 0.0007, PPMC calculated at the best-before date), indicating that the higher the hue angle value, the lower the associated standard deviation. No significant correlation was observed between the standard deviation and the mean of the chroma. These results show that the most heterogeneous Munster cheeses are the darkest with orange-red colors, and the most homogeneous the brightest with yellow-orange colors. 

### 3.3. K-means Clustering of the PDO Munster Cheese Rinds

**Figure 3:**
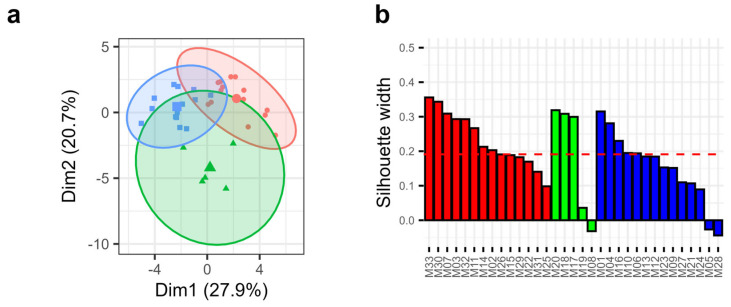
K-means clustering. (**a**) Cluster plot obtained by PCA analysis representing the clusters identified by K-means clustering. The cheeses from clusters 1, 2, and 3 are represented by red points, green triangles, and blue squares, respectively. The center of gravity of each group is represented by a bigger symbol. The circles represent the 95% confidence intervals of each cluster. (**b**) Silhouette width for each of the 33 investigated cheeses (M1 to M33). The cheeses from clusters 1, 2, and 3 are represented in red, green, and blue, respectively.

The evolution of the mean and the standard deviation of the luminance, the chroma, and the hue angle, monitored over time, were used to classify cheeses according to their mean color and color heterogeneity. K-means clustering performed with the color data allowed delineating three clusters (Figure 3a). Cluster 1, 2, and 3 respectively contained 14, 5, and 14 cheeses (Figure 3a). PCA analysis revealed that the first component, which explains 27.9% of the variability, mainly segregates clusters 1 and 2, while the second component, which explains 20.7% of variability, segregates clusters 1 and 2 from cluster 3 (Figure 3a). The groups were rather well segregated since the majority of cheeses had a positive silhouette width except for three cheeses, M08, M05, and M28, for which the value was negative (Figure 3b), indicating that these cheeses were not strongly associated with their group. No significant relationship was observed between the groups and the manufacturing plant, or the effect of pasteurization compared to raw milk. These results show that the variability of Munster cheese color segregates this PDO cheese into three subgroups.

### 3.4. Color Properties of the Three Clusters of Munster Cheeses

**Figure 4:**
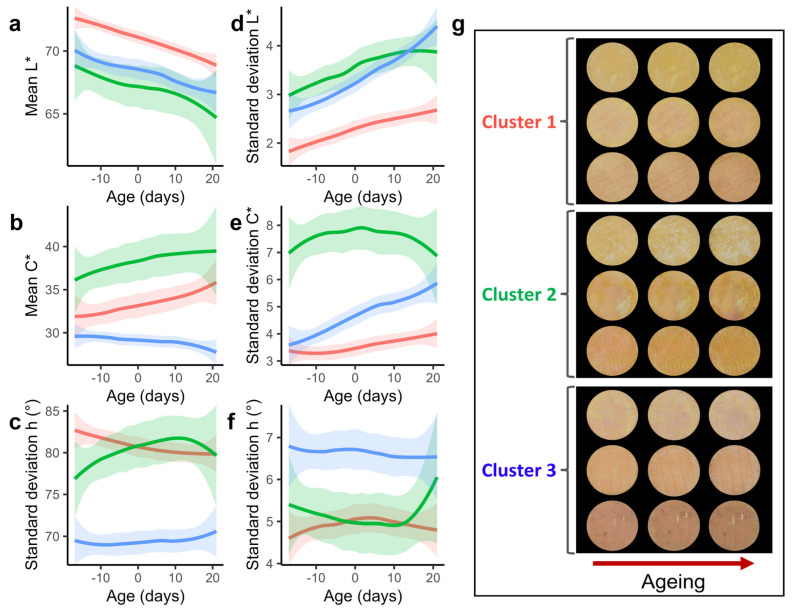
Color properties of the three clusters of cheeses (M1 to M33). The mean (**a**–**c**), the standard deviation (**d**–**f**) of the luminance L* (**a**,**d**), the chroma C* (**b**,**e**), and the hue angle h (**c**,**f**) are represented for the three cheese clusters 1, 2, and 3 in red, green, and blue, respectively. The shaded areas (**a**–**f**) represent the 95% confidence intervals. (**g**) Representative examples of cheeses from the three clusters. For each cheese, three images, displayed horizontally, are shown. The images on the left and on the right were taken at the beginning and at the end of the experiment, respectively, and the images in the middle were taken at the best-before date.

In all groups, there was a statistically significant drop in mean luminance (*p* < 0.05) and an increase in luminance standard deviation over time (*p* < 0.05), showing that all cheeses tended to become darker and more heterogeneous with age (Figure 4a,d,g). However, cheeses from cluster 1 were brighter than the others at all times and less heterogeneous (Figure 4a,d,g, *p* < 0.05). In terms of saturation, an increase in the mean was observed for clusters 1 and 2, although this increase was only significant for cluster 1 (Figure 4b, *p* < 0.05). From the best-before date (age 0) to the end of monitoring, cluster 3 was significantly characterized by the lowest mean color saturation (lower C*) (Figure 4b, *p* < 0.05). In terms of chroma heterogeneity, all clusters were different: cluster 1 had the lowest standard deviation of the chroma, cluster 2 the highest, and cluster 3 was characterized by an increase in the standard deviation, indicating that cluster 1 was the most homogeneous, cluster 2 the most heterogeneous, and that the chroma heterogeneity of cluster 3 increased over time (Figure 4e, *p* < 0.05). Cheeses from cluster 3 had the lowest hue angle and were therefore more orange-red than cheeses from clusters 1 and 2 (Figure 4c, *p* < 0.05). Cheeses from cluster 3 had the highest hue angle heterogeneity regardless of the age (Figure 4f, *p* < 0.05). These results show that Munster cheeses from cluster 3 with orange-red colors had more hue heterogeneity, whereas the yellow-orange colors of clusters 1 and 2 cheeses were more homogeneous (Figure 4g). Overall, these results show that all cheeses became darker over time. Cheeses from cluster 1 were the brightest; they were characterized by a yellow-orange color that became more intense over time and were the less heterogeneous for the three color variables. Cheeses from cluster 2 were also yellow-orange, but they were characterized by a heterogeneous color saturation. Cheeses from cluster 3 were characterized by a heterogeneous orange-red color with a low color saturation that became heterogeneous over time.

### 3.5. Community Structure of Rinds with Different Levels of Color Heterogeneity

**Figure 5:**
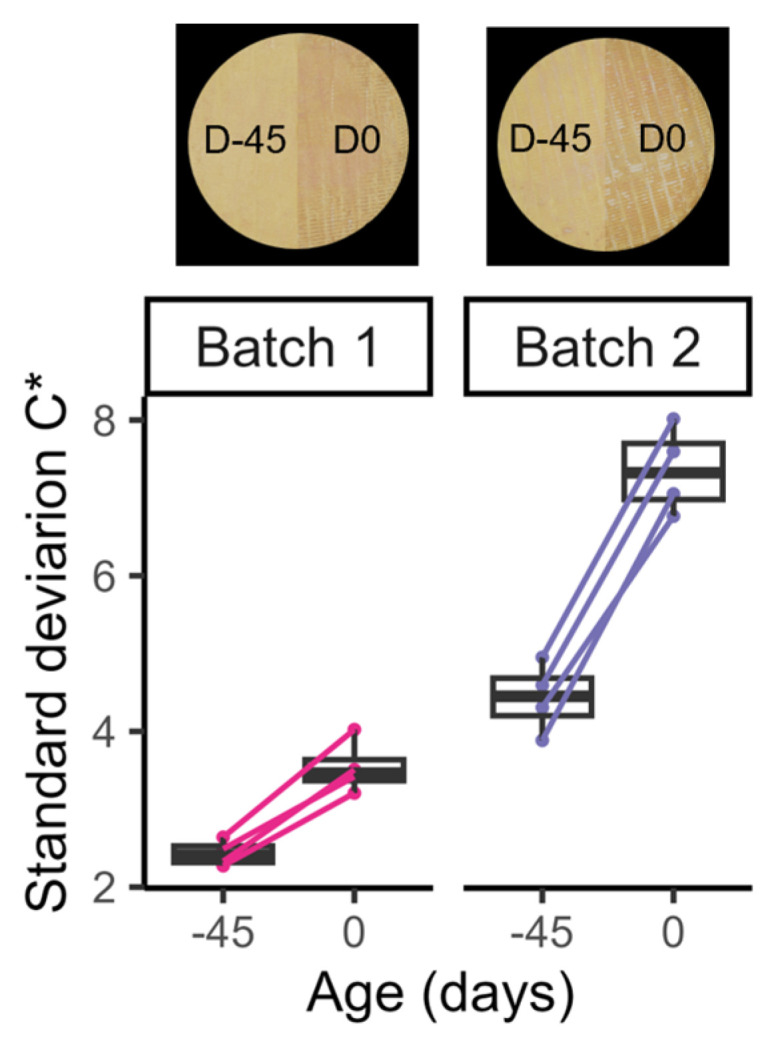
Chroma heterogeneity of the cheese rinds of batches 1 (cheeses M34 to M37) and 2 (cheeses M41 to M44). Boxplot representing the standard deviation of the chroma of the cheese rinds of batches 1 and 2 (from M34 to M37, and from M41 to M44, respectively) at 45 days before (D-45) and at the best-before date (D0). Photographs of one cheese from each batch are presented.

The rind of eight Munster cheeses from two different batches produced in the factory 1 were monitored at two time points: immediately after packaging (45 days before the best-before date; D-45), and at the best-before date (D0). To do so, one side of each cheese was used for analysis at both points in time: one half of the investigated side was investigated at D-45, the other half at D0 (Figure 5, cheese photos). Image analysis revealed that cheeses from both batches were characterized by a significant increase in the chroma standard deviation during storage (Figure 5, *p* < 0.05). This increase was markedly higher for the cheeses from batch 2 (Figure 5). Therefore, these results show that at all times, the cheeses in batch 1 were relatively homogeneous, while those in batch 2 were more heterogeneous.

In batch 1, the counts of bacteria and fungi were slightly lower at D0 compared to D-45 (Figure S1a,b). In batch 2, no difference was observed for the counts of bacteria and fungi between the two time points (Figure S1a,b). Overall, bacteria and fungi approximately reached 3.16 × 10^10^ CFU/g and 3.98 × 10^9^ CFU/g, respectively. No marked differences in the counts of microorganisms were observed between the two batches and between the two time points. 

**Figure 6:**
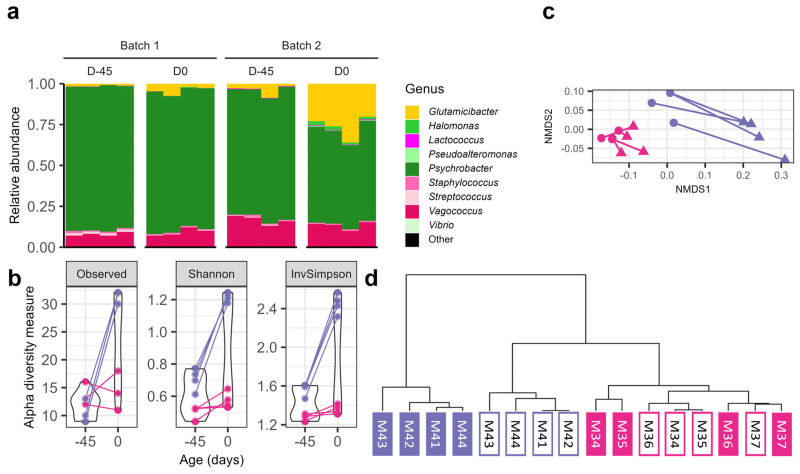
Bacterial community structure analysis of cheese rinds of batches 1 (cheeses M34 to M37) and 2 (cheeses M41 to M44). Cheese rinds of batches 1 and 2, which exhibited homogeneous and heterogeneous colors, respectively, were analyzed 45 days before the best-before date, i.e., immediately after packaging (D-45) and at the best-before date (D0). (**a**) Composition bar plots. The nine major OTUs are represented in different colors. (**b**–**d**) Samples from batch 1 and 2 are represented in pink and violet, respectively. (**b**) Violin plots representing the alpha diversity. (**c**) Non-metric dimensional scaling (NMDS) of samples based on weighted UNIFRAC distance. Points and triangles represent the samples taken at D-45 and D0, respectively. Samples taken from the same cheese at the two time points are connected by a solid line. (**d**) Hierarchical clustering of samples based on weighted UNIFRAC distance and using the centroid-based method ward.D2. Samples taken at D-45 and D0 are represented as empty and filled squares, respectively.

The amplicon-sequencing analyses of the V3–V4 16S RNA gene region showed the presence of three major genera in all conditions: *Glutamicibacter*, *Psychrobacter*, and *Vagococcus,* which were not deliberately inoculated in cheeses (Figure 6a). Cheeses from batch 2, at D-45 as well as at D0, contained a higher relative abundance of *Vagococcus* (16.7 ± 2.7% at D-45 and 13.3 ± 2.4% at D0) compared to cheese from batch 1 (8.3 ± 1.1% at D-45 and 9.6 ± 2.3% at D0) (Figure 6a). In both batches, the relative abundance of *Glutamicibacter* was higher at D0 compared to D-45, and reciprocally the relative abundance of *Psychrobacter* was lower (Figure 6a). However, the increase in relative abundance of *Glutamicibacter* was markedly higher in batch 2 (from 3.5 ± 3.2% at D-45 to 25.8 ± 6.9% at D0) compared to batch 1 (from 0.7 ± 0.4% at D-45 to 3.6 ± 2.5% at D0), and reciprocally, markedly lower for the decrease in *Psychrobacter* (batch 2: from 77.8 ± 2.4% at D-45 to 57.1 ± 4.1% at D0; batch 1: from 88.1 ± 1.2% at D-45 to 85.3 ± 1.7% at D0) (Figure 6a). At D-45, the richness was not different between the two batches and ranged between 9 and 16 Operational Taxonomic Units (OTUs) (Figure 6b, “Observed”). By contrast, at D0, the richness ranged between 11 and 18 OTUs for batch 1, and between 30 and 32 OTUs for batch 2, showing that the richness of batch 2 was markedly higher than that of batch 1 at the best-before date (Figure 6b, “Observed”, *p* < 0.05). The Shannon and InvSimpson α-diversity indices for batch 2 were always higher than for batch 1, and were also markedly higher for batch 2 at D0 (Figure 6b, “Shannon”, “InvSimpson”, *p* < 0.05). The amplicon sequencing of the fungi ITS marker revealed that the α-diversity of batch 2 was higher than that of batch 1 (*p* < 0.05), with little variation for both batches during storage (Figure S2a,b). The increase in diversity occurred within the *Dipodascaceae* family (batch 2: from 8.3 ± 3.9% at D-45 to 13.2 ± 5.8% at D0; batch 1: from 0.0 ± 0.0% at D-45 to 0.1 ± 0.1% at D0) and was attributed to yeasts from the *Galactomyces* or *Geotrichum* genera. In both batches and ages, *Debaryomycetaceae* was the family with the highest relative abundance (batch 2: from 91.6 ± 3.9% at D-45 to 86.7 ± 5.8% at D0; batch 1: from 100.0 ± 0.0% at D-45 to 99.9 ± 0.1% at D0). Overall, these results revealed major differences in the microbiota structure between these two batches and upon cheese storage from the packaging until the best-before date. More specifically, rinds with heterogeneous colors exhibited higher microbial diversity. These results also show that the gain in diversity during storage was greater for cheese rinds of heterogeneous color.

Non-metric multidimensional scaling (NMDS) ordination based on weighted UNIFRAC dissimilarity revealed that cheese rinds from batch 1 presented a similar community structure that changed little during storage (Figure 6c). By contrast, at D-45, rind communities of batch 2 were distant from communities of batch 1, as well as dispersed from each other. In addition, in batch 2, the communities at D0 were distant from the ones at D-45, implying that the microbiota of this batch highly evolved during storage (Figure 6c). Hierarchical clustering confirmed that all the communities of batch 1, including D-45 and D0, were highly similar to each other, as expected for communities that change little over time (Figure 6d). The rind communities from batch 2 at D-45 were closer to the communities of batch 1 than the communities of batch 2 at D0 (Figure 6d). These latter communities were indeed highly distant from all the others (Figure 6d). The Adonis test revealed that the batch of origin and the age explained 49% and 26% of variability, respectively. These results revealed that the cheeses with heterogeneous rind color at the packaging stage were characterized by important changes in their microbial community structure during storage.

### 3.6. The Influence of Salt and pH on Color Heterogeneity

The pH and salt concentration are important factors in cheese ripening and could therefore be related to color development. The salt concentration was higher for batch 1 compared to batch 2 (Figure S3a, *p* < 0.05). More precisely, at D-45, the salt concentration for batches 1 and 2 was 0.68 ± 0.08 and 0.52 ± 0.02 in arbitrary units (AU), respectively (Figure S3a, *p* < 0.05). Similarly, at D0, it was 0.50 ± 0.06 and 0.40 ± 0.02 AU for batches 1 and 2, respectively (Figure S3a, *p* < 0.05). In addition, the pH of batch 2 at D0 (7.45 ± 0.05) was significantly higher than that of batch 1 (6.91 ± 0.11) (Figure S3b, *p* < 0.05). It may be noted that the pH was negatively correlated with the salt concentration at D-45 (r = −0.84, *p* = 0.008 PPMC) (Figure S4a) as well as at D0 (r = −0.78, *p* = 0.022 PPMC) (Figure S4b). These results show that the two batches exhibiting different color heterogeneity properties were characterized by different salt concentrations and final pH. 

### 3.7. Link between Community Structure and Heterogeneity at the Intra-Cheese Spatial Scale

**Figure 7:**
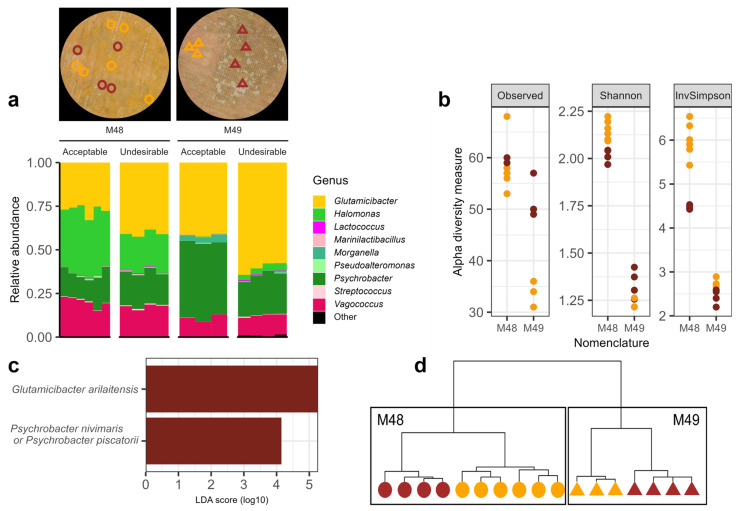
Bacterial community structure analysis of cheeses M48 and M49. The intra-cheese spatial variability of the community structure was investigated by analyzing between three and six samples, each covering approximately 0.2 cm^2^, on zones with acceptable and undesirable colors. (**a**) Images of the cheeses M48 and M49 photographed the day of sampling. The sampled zones are indicated with circles (M48) and triangles (M49), in orange and brown for acceptable and undesirable zones, respectively. Below the images are presented composition bar plots of the samples where the nine major OTUs are represented in different colors. (**b**) Plots showing the alpha diversity. Yellow and brown points represent samples taken from patches of acceptable and undesirable colors, respectively. (**c**) Linear discriminant analysis effect size (LEfSe) using an LDA score cutoff of 4, performed on the microbial relative abundance data representing enrichment in undesirable color. (**d**) Hierarchical clustering of samples using a centroid-based method (ward.D2) and based on Bray–Curtis distances. Samples taken in acceptable and undesirable color zones are represented in yellow and brown, respectively. Filled circles and triangles represent the samples taken from the surface of cheeses M48 and M49, respectively.

In some cases, color variability leads to the appearance of patches with undesirable colors. For instance, in factory 1, cheeses with brown patches are regularly obtained. To investigate this particular color heterogeneity, two cheeses, M48 and M49, with such undesirable brown patches and acceptable orange patches were analyzed by metabarcoding (Figure 7a). Among the dominant bacterial OTUs, the three genera *Glutamicibacter* (32.1 ± 7.4% for M48, and 51.7 ± 10.2% for M49), *Psychrobacter* (17.1 ± 3.2% for M48, 31.5 ± 11.1% for M49), and *Vagococcus* (19.2 ± 2.7% for M48, 11.2 ± 1.4% for M49) were found in all cheeses whatever the zone. In addition, for cheese M48, the relative abundance of *Halomonas* OTU was high (30.0 ± 8.1%) by contrast with cheese M49, where *Halomonas* were found in low relative abundance (2.1 ± 1.8%) (Figure 7a). For M48, the relative abundance of *Glutamicibacter* was higher in undesirable patches (40.2 ± 1.7%) than in acceptable patches (26.7 ± 3.1%), at the expense of *Halomonas* (21.2 ± 0.7% and 35.9 ± 3.8% in undesirable and acceptable patches, respectively). Similarly, for M49, the relative abundance of *Glutamicibacter* was higher in undesirable patches compared to acceptable ones (59.6 ± 3.2% and 41.1 ± 0.8%, respectively), except that in this case, it was reciprocally linked to the lower relative abundance of *Psychrobacter* (22.7 ± 2.1% and 43.2 ± 1.7% in undesirable and acceptable patches, respectively). Among fungi, the dominant OTUs belonged to the family *Debaryomycetaceae* (85.4 ± 4.8% in M48, and 72.6 ± 15.8% in M49) and *Dipodascaceae* (14.6 ± 4.8% in M48, and 21.6 ± 12.8% in M49) (Figure S5a). For all bacterial alpha diversity metrics, M49 tended to have lower values than M48, with statistical differences for the Shannon and Inverse-Simpson indices (Figure 7b). However, for both bacteria and fungi, there was no clear link between the color and the alpha diversity (Figure 7b and Figure S5b).

Hierarchical clustering based on Bray–Curtis dissimilarity revealed that samples from cheeses M48 and M49 clustered into two main branches (Figure 7d) that explained 70% of community structure variability. Each of these branches itself splits into two branches in which the samples are grouped by color, explaining 12% of the variability. An LEfSe analysis revealed, as expected, that a higher abundance of *G. arilaitensis* is linked to undesirable brown colors. Importantly, although the overall relative abundance of *Psychrobacter* was lower in undesirable patches, the LEfSe analysis also revealed a higher abundance of *Psychrobacter nivimaris* or *Psychrobacter piscatorii* in undesirable brown color patches (Figure 7c, LDA score [log 10] > 4). More precisely, in M48, a higher relative abundance of *P. nivimaris/piscatorii* was observed in undesirable patches compared to acceptable ones (2.8 ± 1.8% and 0.1 ± 0.1%, respectively), and similarly in M49 (0.6 ± 0.5% and 0.0%, respectively). This result indicates that these species were the most differentially abundant bacterial taxa in the patches with undesirable colors compared to the ones with acceptable colors. 

## 4. Discussion

In this study, the color diversity of the PDO Munster cheeses was investigated with a special emphasis on heterogeneity and the link with biotic and abiotic factors. To do so, the evolution of the color of 33 Munster cheeses was monitored during storage. In addition, two batches of cheeses produced in the same factory were investigated for a possible link between the rind color heterogeneity and the microbiota, as well as the pH and the salt concentration. Furthermore, two cheeses exhibiting multiple undesirable brown color patches were studied in terms of microbiota composition. 

Despite the importance of cheese color in consumer appreciation of cheese quality, few studies have been carried out to document cheese color and understand the underlying mechanisms. The color of cheeses is classically investigated by using spectrocolorimetry [3]. This approach allows the measurement of the overall color of zones of a fixed surface dimension, which depends on the measuring instrument. Typically, the color of the rind of smear-ripened cheeses was previously measured with a spectrocolorimeter covering zones of several square centimeters, which is not well adapted to the analysis of the spatial heterogeneity of the color [3]. In this study, image analysis was performed thanks to a standardized photographic approach allowing us to analyze the color at the pixel scale. Thus, between 10 and 14 measurements per cheese were previously performed with a spectrocolorimeter [3], while in our study each cheese was segmented into more than 90,000 pixels, resulting in a much higher resolution for color dispersion investigation. Photographic imaging is an alternative approach that has been successfully used to investigate the color of food products [51], including mozzarella cheese [52]. This system can provide a detailed characterization of color uniformity at a fine spatial scale, quantifying surface characteristics, non-uniform color distribution, and defects [31,51]. A pixel corresponds to a surface area of the order of one square millimeter in our study, giving rise to a sufficiently high spatial resolution for the analysis of color heterogeneity.

To our knowledge, this study is the first to describe the evolution of the heterogeneity of cheese rind color. A few studies have investigated the mean color of cheeses [53,54], including one about smear-ripened cheeses [3]. The previously described mean colorimetric parameters of Munster cheese (a* values from 7 to 17 and b* values from 23 to 47) were similar to those observed in this study (a* from 0.6 to 14.9 and b* from 20.9 to 51.9), corresponding to yellow to orange-red colors, in good agreement with the expected range from ivory-orange to orange-red [2]. Our study showed that the standard deviation of the hue angle and the luminance is negatively correlated with their respective mean. This result revealed that the level of heterogeneity of the rind is related to its color characteristics, suggesting that this could be used as a technological lever to control the color heterogeneity of the rind. Orienting the ripening of the cheese towards the development of a luminous and yellow rind would lead to limited color heterogeneity. Alternatively, encouraging the development of a dark orange-red rind could favor the appearance of heterogeneity. 

Our results revealed that PDO Munster cheeses can be divided into three groups based on the evolution of color mean and heterogeneity. The first group was the most luminous and the most homogeneous. These cheeses tended to be yellow and became more orange with time. The cluster 2 had few representatives (five Munster cheeses compared to 14 for the other groups). Cheeses from cluster 2 were darker than those from cluster 1 and had heterogeneous luminance values. They also had the highest and most heterogeneous color saturation values (C*), whatever the age. Cluster 3 was quite similar to cluster 2 regarding luminance and luminance heterogeneity. However, the cheeses of this cluster had the lowest color saturation and hue angle, which corresponded to the more pronounced reddish color compared to other groups. It was also the group with the most heterogeneous hue angle. To the best of our knowledge, the microbiological and molecular basis of the evolution of color mean and heterogeneity over time has not been described in the literature. It can be speculated that this increase may be due to an increase in pigment concentration or a change in molecular composition and/or structure.

By contrast to this study, two groups of Munster cheeses seemed to be distinguishable from previous data [3]. These differences are probably related to the color variables used for clustering. In the study of Dufossé et al., two clusters can be observed in the a*b* space [3]. In our study, the K-means clustering algorithm was used with a total of 24 variables, including the evolution of the mean and the standard deviation of the three variables L*, C*, and h. The integration of this large number of variables has probably made it possible to provide the discriminant data enabling these three sub-groups of Munster cheese to be distinguished. 

Cheese color is an important factor in consumer perception. For Cheddar, consumers prefer light orange cheeses to dark orange or white varieties. In addition, color influences how consumers perceive other characteristics of the cheese: light orange and white cheeses are better associated with naturalness than orange cheeses [1]. For Munster PDO cheese, it would be interesting to study consumers’ perceptions of the three clusters found in our study, and more specifically whether they have a preference for clusters and whether they associate clusters (color and heterogeneity) with different characteristics such as taste of the Munster cheese type (industrial vs. artisanal, pasteurized vs. raw milk).

Metabarcoding analysis revealed a low diversity of fungi with the presence of two major families: *Dipodascaceae* and *Debaryomycetaceae*, which likely corresponded to the microorganisms intentionally added as cultures, i.e., *Geotricum candidum* and *Debaryomyces hansenii*, respectively. Similar results were obtained for Epoisses and other smear-ripened cheeses [55,56], suggesting that adventitious fungal species do not seem to be common in those cheeses. 

Three major bacterial genera were found in all cheese rinds: *Glutamicibacter* and *Vagococcus* (Gram-positive)*,* and *Psychrobacter* (Gram-negative). *Glutamicibacter arilaitensis* is not included in the current QPS list [57] but is present in the inventory of microbial food cultures with safety demonstration in fermented food products of the International Dairy Federation (IDF) [58]. Strains of this species are commercially available for use in cheese ripening [59]. It can contribute to color and aroma development [21,22]. *Vagococcus* is not present either in the current QPS or in the IDF list [57,58]. These lactic acid bacteria are poorly described in the literature but were previously described as members of cheese microbiota [26]. *Psychrobacter* spp. are frequently found in cheeses [26,55,60] and *Psychrobacter celer*, which is present in the IDF list [58], can produce aromatic volatile compounds in cheeses [61]. These microorganisms were not deliberately inoculated during cheese processing. These genera were also previously found in Munster cheeses, although they were not the most abundant [26]. It can be noted that *Brevibacterium*, which was intentionally inoculated as a culture in the cheeses produced by factory 1 investigated in this study, was detected in low relative abundance (at most, 0.25%). This was repeatedly described for other smear-ripened cheeses, suggesting that *B. linens* is not well adapted to this type of cheese [15,55,62,63,64]. This suggests that *B. linens* is not adapted to the abiotic conditions of the studied Munster cheeses. Alternatively, these bacteria could have been excluded by adventitious flora through competition or inhibition [62]. More interestingly, and similarly to our results, it has been shown in Epoisses cheese that the relative abundance in *G. arilaitensis* increases, and reciprocally, that the proportion of *Psychrobacter* decreases during storage at 4 °C. These observations suggest some common pattern of community dynamics for smear-ripened cheeses during storage.

The investigation of two cheese batches produced in the same factory, which differed by their level of color heterogeneity, revealed that the most heterogeneous cheeses were characterized by different community structures. In addition, a microbiome spatial analysis revealed a significant association between the color of the rind and the structure of bacterial communities. The utilization of coloring additives is not allowed in this PDO cheese, implying that the color of the rind of Munster cheese is mainly due to microbial activity. Interestingly, a significant association between the spatial distribution of the color and the relative abundance of *G. arilaitensis* was observed. The species produces β-caroten and porphyrins that can confer a color to the rind ranging between yellow, orange, brown and red [7,9,18]. It can be hypothesized that the color heterogeneity of the rind of Munster cheeses is related to the pigment production properties of *G. arilaitensis*. Spatial fluctuation in the relative abundance of *Glutamicibacter* could have led to the overproduction of pigments. Alternatively, although an overall decrease in the relative abundance of the genus *Psychrobacter* was observed in zones with undesirable colors, an increase in the relative abundance of *P. nivimaris/piscatorii* was also observed in these zones. It could be hypothesized that *P. nivimaris/piscatorii* interact with the coloring properties of *G. arilaitensis*, since it has been described that biotic interactions may affect the coloring properties of *G. arilaitensis* [14,18]. *P. nivimaris/piscatorii* could also cause color defects independently of *G. arilaitensis*. It has been described that *Psychrobacter* species can cause color defects in surface-ripened cheese by producing purple-red pigments [65]. 

The spatial heterogeneity of microbial communities of cheeses is poorly described, by contrast with other anthropogenic systems such as cultivated lands. Human activities often imply biotic homogenization of ecosystems. For instance, the conversion of lands for agricultural purposes induces taxonomic homogenization of soil microbial communities [66]. Biotic homogenization leads to a decrease in beta diversity between sites, and reciprocally, a higher heterogeneity leads to an increase in beta diversity [67]. Consistently, the analyses of heterogeneous cheeses at a small spatial scale performed in the present study, where samples covered approximately 0.2 cm^2^, have shown that the beta diversity was significantly higher between sites of different colors compared to sites of similar colors. Also, the analysis of cheeses at higher spatial scale, where sample covered half a side of cheese, representing approximately 284 cm^2^, revealed that rinds with heterogeneous colors exhibited higher microbial alpha diversity. This increase in alpha diversity was indeed expected since this sampling scale encompasses the different community variations that can occur on half a cheese side. These results highlight the importance of spatial scaling, which is well-recognized in the field of landscape ecology [68].

Salt and pH are critical parameters in cheese manufacturing [69]. In this study, the pH of the rind significantly increased during storage, which has also been observed for the smear-ripened Époisses cheeses [55]. A decrease in salt concentration was also observed during storage of Munster cheeses. During cheese making, dry salt is added on the surface of the curd, and it is described that a slow diffusion of the salt from the surface to the core takes place during ripening, leading to a decrease in salt concentration on the surface [70]. Salt concentration can have a strong negative impact on the growth of microorganisms, and thereby on the pH increase through the ripening activity of the microorganisms in the rind. The lower salt concentration of batch 2 compared to batch 1 could have led to a higher ripening activity and therefore to the higher pH observed in batch 2 compared to batch 1. Interestingly, the rinds of cheeses from batch 2 were characterized by a higher heterogeneity than those of the cheeses from batch 1, suggesting that lower salt concentration and/or higher pH favor the development of rind color heterogeneity. This suggests that salt could be used as a technological lever to control the degree of color heterogeneity of the rind of Munster cheeses.

## 5. Conclusions

Cheeses are complex ecosystems in which the communities are spatially structured. This spatial organization and its functional consequences are poorly understood. This study has shown the color heterogeneity of Munster cheese at the temporal scale and, more importantly, at multiple spatial scales. It has shown the relationship between color heterogeneity and the microbial community structure as well as abiotic factors, and thus revealed that cheeses can be very interesting models to study microbial landscapes, which have been largely overlooked so far. Future studies that better account for this ecosystem heterogeneity could significantly contribute to a better understanding and control of the functioning of these food ecosystems. More specifically, the strains colonizing these cheeses could be isolated, and synthetic ecology experiments could be carried out in model cheeses to understand the underlying ecological mechanisms involved in color heterogeneity.

## Figures and Tables

**Table 1:** Primers used for PCR.

Target Region	Primer Orientation	Primer Name	Primer Sequence (Targeting Sequence in Bold)	Reference
V3–V4 16S rRNA	Forward	16SV3F-1id	TCGTCGGCAGCGTCAGATGTGTATAAGAGACAGNNNNNN**TACGGRAGGCWGCAG**	[35]
		16SV3F-2id	TCGTCGGCAGCGTCAGATGTGTATAAGAGACAGNNNNN**TACGGRAGGCWGCAG**	[35]
		16SV3F-3id	TCGTCGGCAGCGTCAGATGTGTATAAGAGACAG**TACGGRAGGCWGCAG**	[35]
	Reverse	16SV4R-id	GTCTCGTGGGCTCGGAGATGTGTATAAGAGACAGNNNNN N**TACCAGGGTATCTAATCCT**	[35]
ITS	Forward	ITS1F-1id	TCGTCGGCAGCGTCAGATGTGTATAAGAGACAGNNNNNN**CTTGGTCATTTAGAGGAAGTAA**	[36]
		ITS1F-2id	TCGTCGGCAGCGTCAGATGTGTATAAGAGACAGNNNNN**CTTGGTCATTTAGAGGAAGTAA**	[36]
		ITS1F-3id	TCGTCGGCAGCGTCAGATGTGTATAAGAGACAGNNNN**CTTGGTCATTTAGAGGAAGTAA**	[36]
	Reverse	ITS2R1id	GTCTCGTGGGCTCGGAGATGTGTATAAGAGACAGNNNNNN**GCTGCGTTCTTCATCGATGC**	[37]

## Data Availability

The dataset repository and the analysis code used in this study can be found in the data repository Recherche Data Gouv at https://doi.org/10.57745/QQJBDD (accessed on 17 June 2024).

## Supplementary Materials

The following supporting information can be downloaded at: https://www.mdpi.com/article/10.3390/foods13142233/s1, Table S1: Cheese samples used in this study; Figure S1: Microbial load of cheese rinds of batches 1 (cheeses M34 to M37) and 2 (cheeses M41 to M44). Boxplots representing (a) the bacterial and (b) the fungal enumeration of batches 1 and 2; Figure S2: Fungal community structure analysis of cheese rinds of batches 1 (cheeses M34 to M37) and 2 (cheeses M41 to M44). Cheese rinds of batches 1 and 2, which exhibited homogeneous and heterogeneous colors, respectively, were analyzed 45 days before the best-before date, i.e., immediately after packaging (D-45), and at the best-before date (D0). (a) Composition bar plots. (b–d) Samples from batches 1 and 2 are represented in pink and violet, respectively. (b) Violin plots representing the alpha diversity. (c) Non-metric dimensional scaling (NMDS) of samples based on weighted UNIFRAC distances. Points and triangles represent the samples taken at D-45 and D0, respectively. Samples taken from the same cheese at the two time points are connected by a solid line. (d) Hierarchical clustering of samples using the centroid-based method ward.D2 and based on weighted UNIFRAC distances. Samples taken at D-45 and D0 are represented as empty and filled squares, respectively; Figure S3: Distribution of pH and salt concentration in batches 1 and 2. Each half of the monitored cheese sides of batches 1 and 2 was subjected to 10 measurements of pH and salt concentration at different locations. A mean value was calculated from each set of measurements, giving rise to two mean pH values and two salt mean concentrations for each cheese, one for each time point D-45 and D0. Boxplot representing the mean values of pH (a) and salt concentration (b) of batches 1 and 2. Lines connect the data obtained from the same cheese units; Figure S4: Relationship between pH and salt concentration. Each half of the monitored cheese sides of batches 1 and 2 was subjected to 10 measurements of pH and salt concentration at different locations. A mean value was calculated from each set of measurements, giving rise to two mean pH values and two salt mean concentrations for each cheese, one for each time point D-45 and D0. Plots representing the mean value of pH and salt concentration of the overall eight cheeses of batches 1 and 2 at D-45 and D0 are presented in panels a and b, respectively. The red line represents the model obtained from linear regression of data; Figure S5: Fungal community structure analysis of cheeses M48 and M49. The intra-cheese spatial variability of the community structure was investigated by analyzing between four and six samples, each covering approximately 0.2 cm^2^, on zones with acceptable and undesirable colors. (a) Composition bar plots of the samples. (b) Plots showing the alpha diversity. Yellow and brown points represent samples taken from zones of acceptable and undesirable colors, respectively.
