# Supplementary figures and images for: Deciphering Rind Color Heterogeneity of Smear-Ripened Munster Cheese and Its Association with Microbiota
Source: Foods. 2024 Jul 16;13(14):2233. doi: 10.3390/foods13142233 (PMC11276107; doi:10.3390/foods13142233)

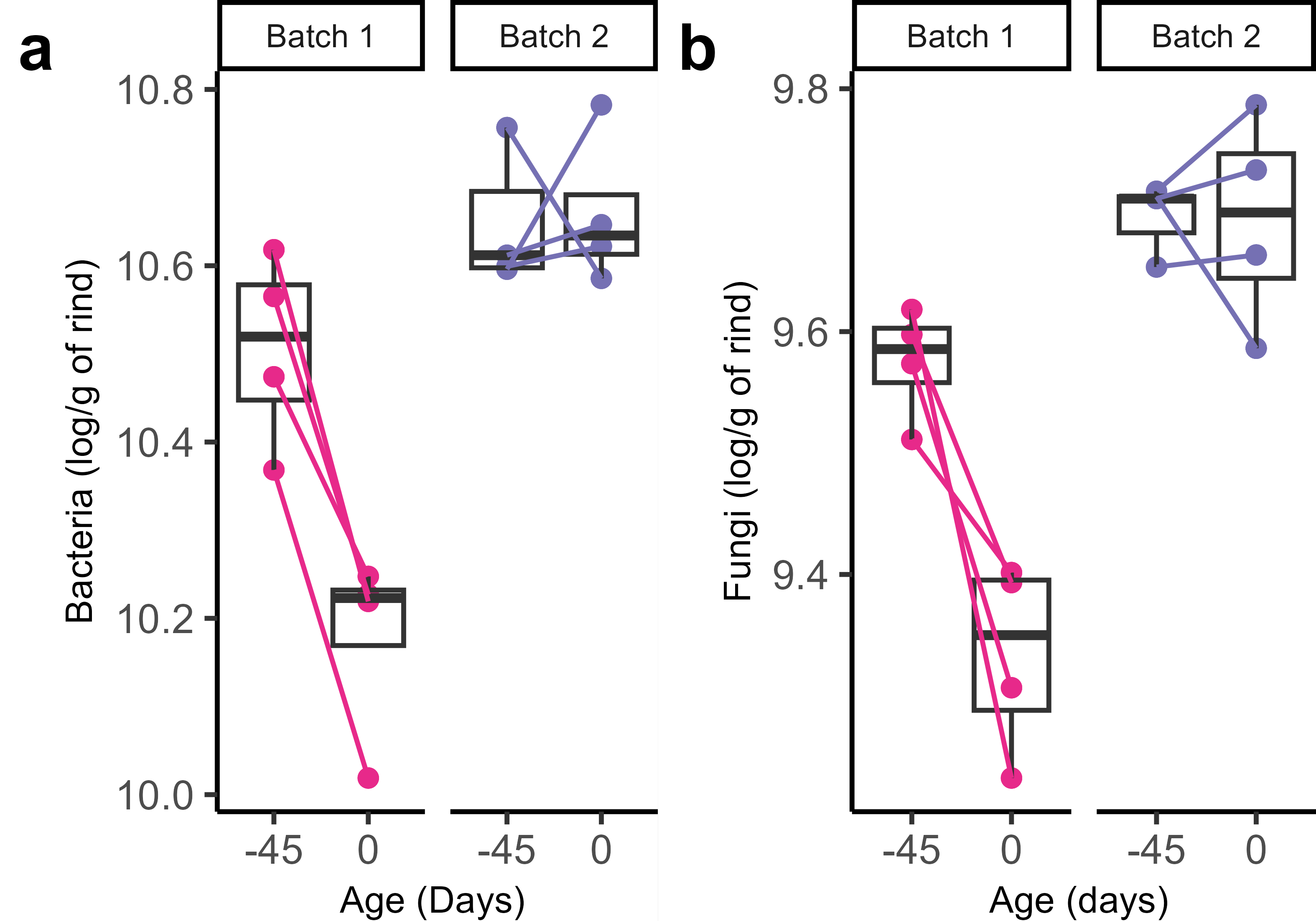

Supplement: Supplementary file 1 [file foods-13-02233-s001.zip › FS1.tiff]

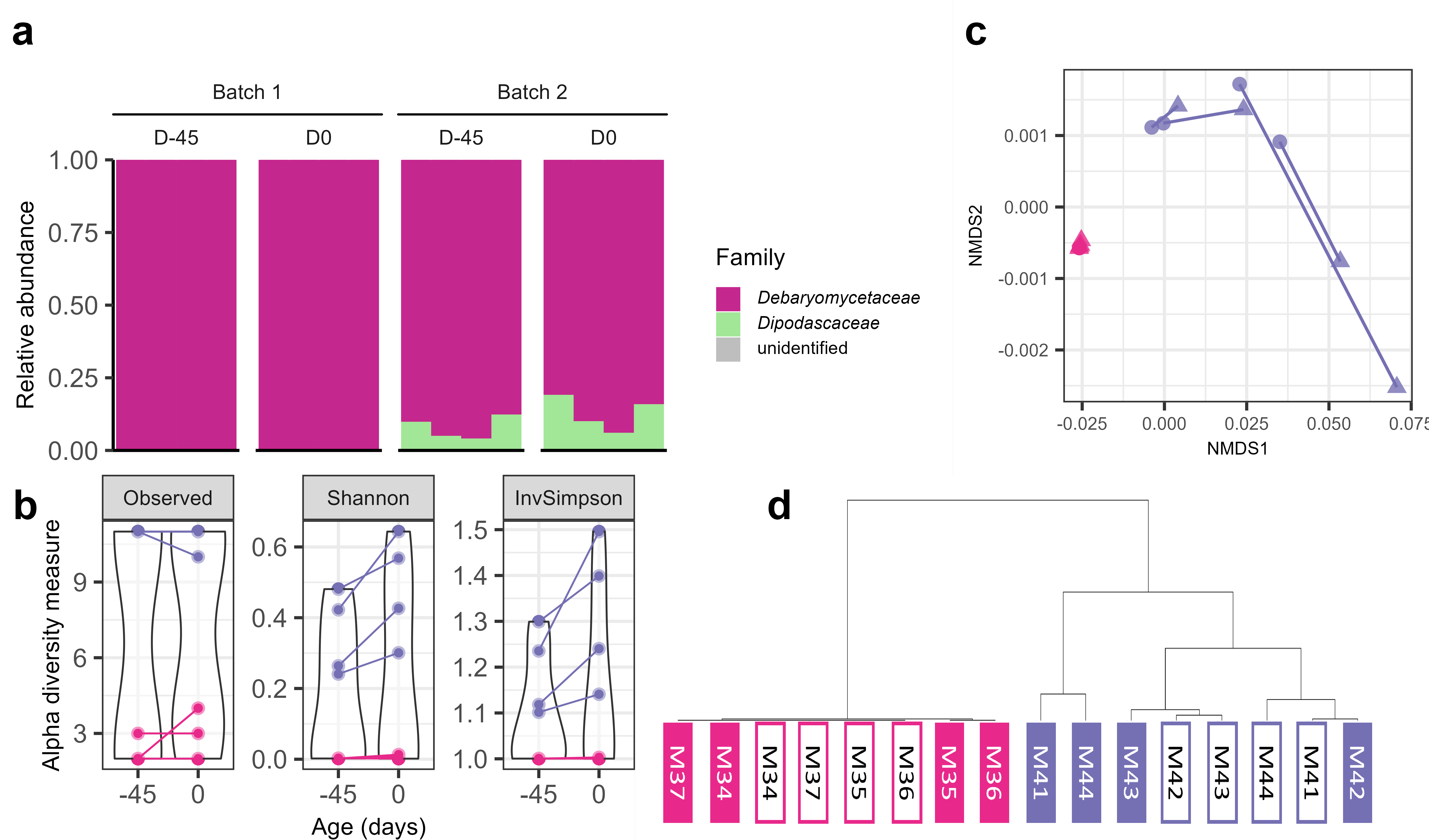

Supplement: Supplementary file 1 [file foods-13-02233-s001.zip › FS2.tiff]

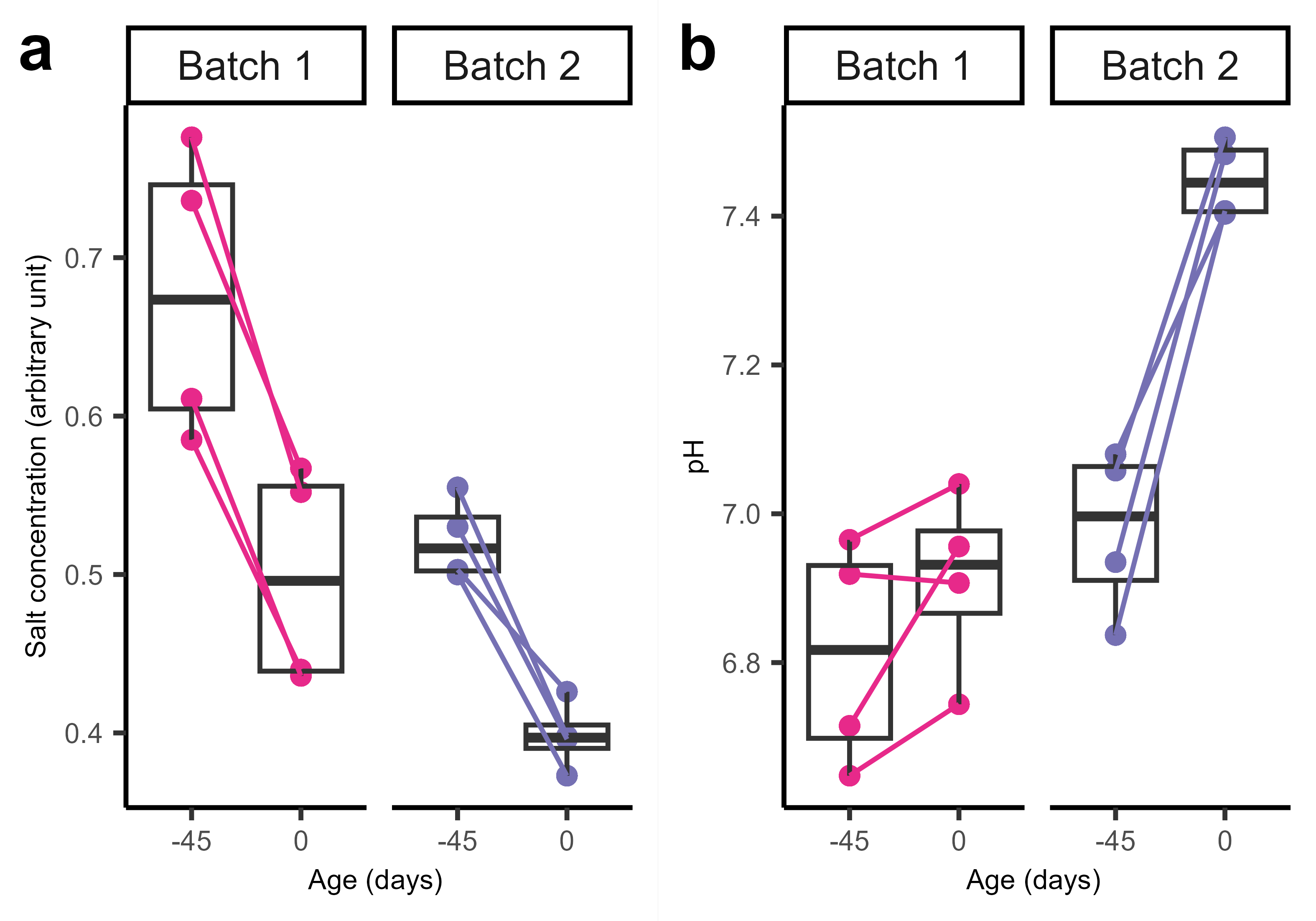

Supplement: Supplementary file 1 [file foods-13-02233-s001.zip › FS3.tiff]

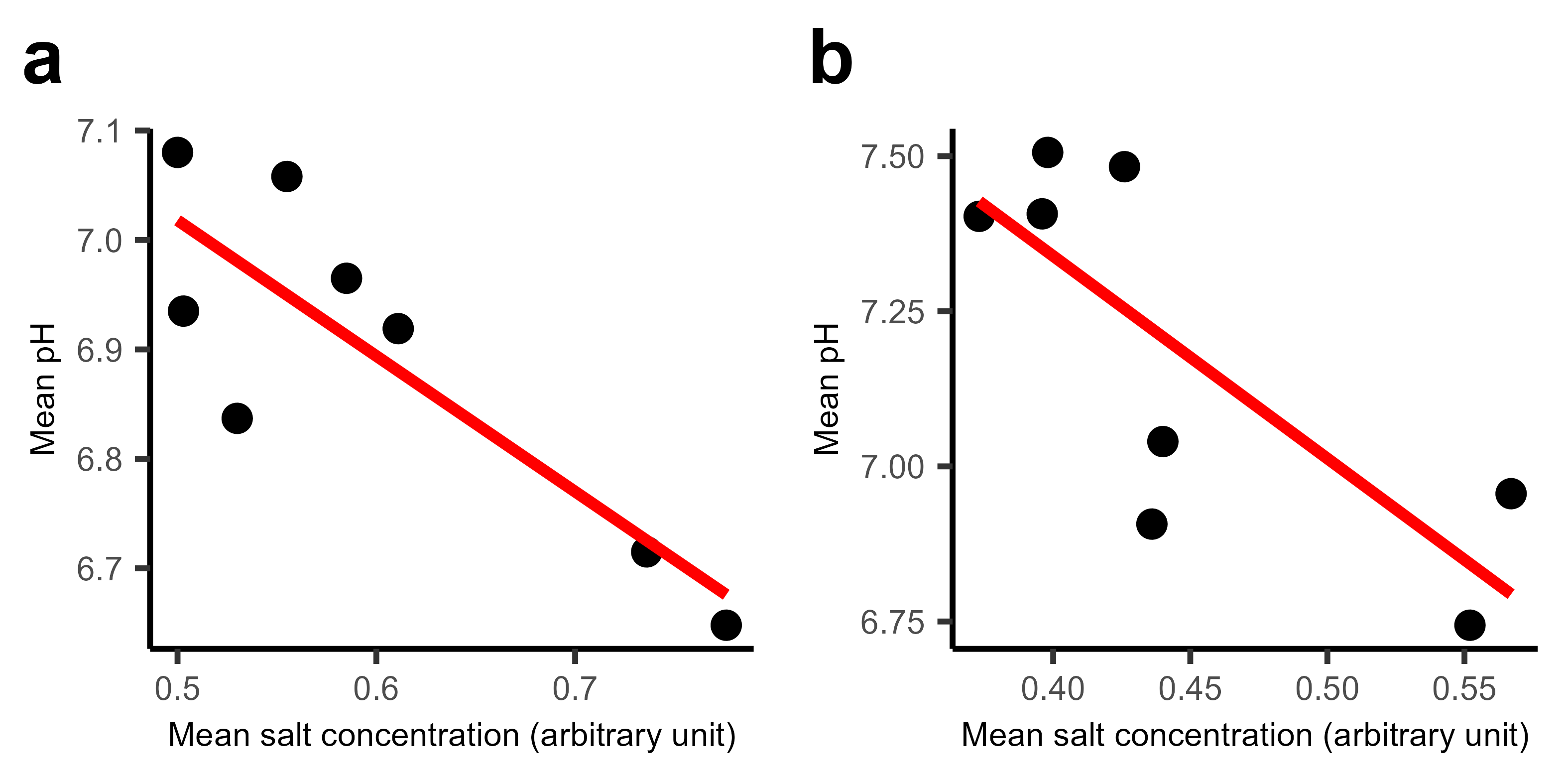

Supplement: Supplementary file 1 [file foods-13-02233-s001.zip › FS4.tiff]

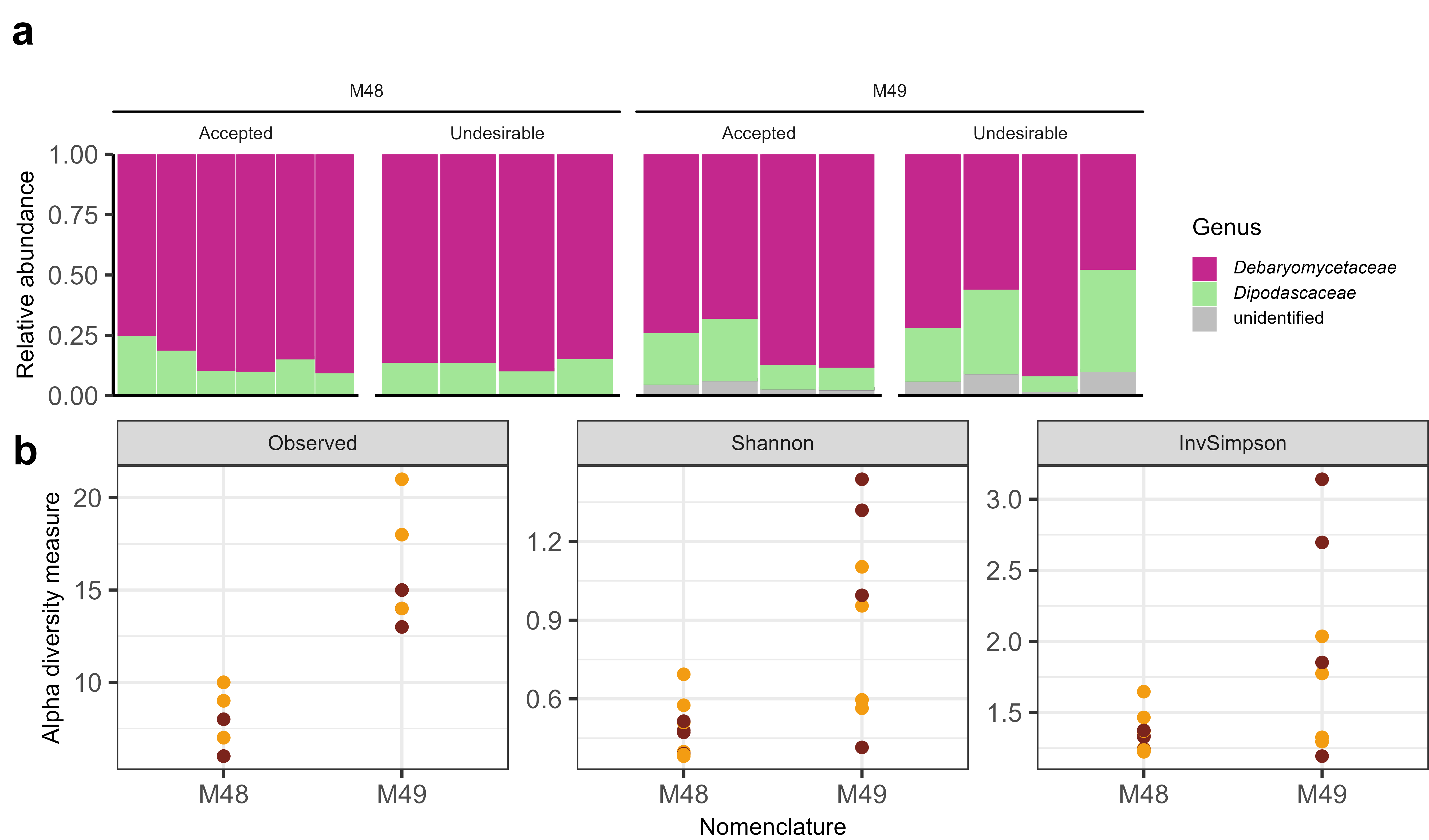

Supplement: Supplementary file 1 [file foods-13-02233-s001.zip › FS5.tiff]
